# Meeting Reports

**DOI:** 10.1155/S1463924680000648

**Published:** 1980


### Meeting Reports

### Microcomputers and chemistry

The inaugural meeting of the Microcomputer and Microprocessor Group of the Royal Society of Chemistry was held at the Imperial College of Science and Technology, London on 10th July 1980. The morning session was preceded by the election of the Committee of the Group.

The first talk, given by Mr James of the Imperial College of Science and Technology Computer Centre, presented a current survey of microcomputers and the criteria which should be applied before purchase. He described the balance of relative costs in running/buying a computer whereby hardware is becoming relatively inexpensive and software is becoming more expensive. The second talk, given by Dr Newton of AERE, Harwell, described how microcomputers could be used for laboratory control applications and data acquisition. Various forms of analogue/digital and digital/analogue convertors were described in detail.

The afternoon session started with two talks on specific applications of microcomputers in the laboratory. The first was given by Dr Goddard of the Imperial College of Science and Technology who described the use of a Research Machines 380Z microcomputer to control an automatic titrator. The second by Mr. Huddleston of AERE, Harwell described the use of a PET microcomputer for the acquisition and processing of data from a nuclear microprobe. The final talk was given by Dr Boswell of the Victoria University of Wellington, New Zealand. The relative merits of microcomputers and mainframe computers were described and the arguments for and against the use of assembly language as opposed to high level language were considered.

Although the venue for this meeting was not particularly suitable, the content was very enjoyable and should ensure the future success of this new group.

*Allan Dennis*

### The 1980 Summer School of Automatic Chemical Analysis

Following the success of 1979, the Summer School of Automatic Chemical Analysis returned to the Chemistry Department of the University College of Swansea. The School, held from the 6th to the 11th July, was organised on this occasion by Professor D Betteridge, Dr P B Stockwell and Mr D G Porter. Comfortable accommodation and good meals were provided at Neuadd Martin, hall of residence. The lecture programme was similar to the successful format of the previous year covering the history of, and modern developments in, automatic chemical analysis, aspects of laboratory management, the interaction of modern electronics with chemical analysis and the automation of some particular analytical techniques. The tutorial sessions generated a considerable amount of discussion and some new ideas were exchanged, the course problem on the ways in which a smoking programme for tar and nicotine yields of cigarettes could be automated generated a number of possibilities, some of particular novelty. The lecturers included Mr T G Alliston, Dr C R Loscombe, Mr D G Porter and Dr P B Stockwell, from the Laboratory of the Government Chemist–Dr J K Foreman from the National Physical Laboratory; Dr D R Deans and Mr D C M Squirrel from ICI; Dr F L Mitchell from the Clinical Research Centre; Mr P G Sanders from St Bartholomew's Hospital; Professor M B Denton from the University of Arizona; Professor D Betteridge from the University College of Swansea; Dr K Stewart from the United States Department of Agriculture; and Mr F B Fearn from Technicon.

*T. G. Alliston*

### Fifth SAC Conference (SAC '80)

During the week 20-26 July 1980, the Analytical Division of the Royal Society of Chemistry held the fifth in the series of triennial international conferences started by the former Society for Analytical Chemistry (SAC) in 1965.

This conference, held at the University of Lancaster, covered a wide range of analytical topics with a notable series of plenary lectures, together with both submitted lectures and posters. In addition, workshop sessions were held in which participants exhibited apparatus constructed and used in their laboratories; several of the Analytical Division's constituent groups organised discussion sessions on topics relevant to their interests; and a fairly sizeable manufacturer's exhibition was put on. There was also a full social programme.

With such a varied programme it would perhaps be expected that there would be much to interest those working in the field of automation. This was only partially true. Certainly some of the contributors to the workshop sessions showed automated apparatus with a spark of originality and the Automatic Methods Group Discussion Session produced a worthwhile discussion on the use of graphical displays in microprocessor controlled instrumentation. Yet relatively few of the 150 odd papers and posters dealt with automation per se. A visit to the manufacturer's exhibition provided perhaps one clue - microprocessor controlled analytical instrumentation is now very much in vogue and one cannot help wondering if automation is not entering a new phase with less emphasis on individual laboratories developing their own automated procedures and instrumentation, and more on them using what is in many ways an excellent range of commercially available equipment. There is no question, of course, that automation has come of age, and that the dedicated work of the pioneers has not been in vain. It would be a pity, however, if this were to be accompanied by a decline in individual innovation. Perhaps I am being unduly pessimistic. I hope so.

Looking briefly at the conference, particularly interesting items at the workshop sessions included a novel microprocessor controlled centrifugal solvent separation system, developed at the Laboratory of the Government Chemist, and based on a combination of angular acceleration and centripetal force to effect a separation of two immiscible solvents absorbed into the pores of a porous nickle-chrome alloy; a microcomputer controlled auto-titrator for the determination of halides, developed by ICI Plastics Division; a display of data acquisition and manipulation devices, based on the use of low-cost computers (PET, APPLE, etc), and shown by the Instrumentation and Applied Physics Division, AERE, Harwell; and a low-cost, 'home-made' computer controlled automated flow injection apparatus for the determination of phosphate, exhibited by Albright and Wilson Limited.

The Automatic Methods Group held an interesting and useful discussion session on the subject 'Exploiting Microprocessors for Graphical Display'. This was chaired by Professor Malmstadt and attended by some 50 people. Professor Malmstadt opened the proceedings with some general comments on the use of microcomputers in analytical instrumentation. He then introduced Dr D. Deans (ICI Petrochemicals Division) who spoke briefly on the types of visual information provided by graphical displays both for the analyst and the laboratory manager. He particularly emphasised the ability to display analogue information and movement. Dr R. Smith (Loughborough University of Technology) then discussed the uses of graphical display in a wide range of spectroscopic techniques, with particular reference to the display of data, results, operating conditions, statistics and reports. In the ensuing discussion topics considered included interfacing problems, the use of 'touch-screens' for diagnostic purposes, voice inputs, etc.

The main paper on automation was presented by Professor Malmstadt in his plenary lecture on 'Analytical Instrumentation for the 1980's' - he discussed how microcomputers have come to play an increasing role in the laboratory, using as examples some of the work undertaken in his own department.

In conclusion, SAC 80 continued the high standards set by its predecessors both in scientific content and in the range of social activities. It continues to provide an excellent triennial opportunity for analytical chemists to meet their colleagues world-wide. Bearing in mind my earlier comments it will be interesting to see the developments in automation and computerisation over the three years before the next SAC Conference. This reviewer hopes that his remarks on the decline in contributions from the individual laboratory in the field of automation will have proved to be too pessimistic.

*Clive J. Jackson*

### New Literature

### Circulators

In a new bulletin, Fisher Scientific highlights three circulators for circulating temperature-controlled fluids through external apparatus. The model 73 provides ±0.1°C control within its ambient to 150°C temperature range. This immersion circulator can be clamped to the edge of a tank, aquarium or jar-style bath. Designed for close temperature control, the model 80 features an insulated one gallon equilibration bath. Within its ambient to 150°C temperature range, control accuracy is claimed to be ±0.003°C. This circulator can be connected to an auxiliary coolant source for operation at temperatures from below 0°C. Designed for low temperatures model 90 heats or cools small flasks and tubes to temperatures between -15° and 100°C with ±0.02°C accuracy. It is equipped with a built-in refrigeration unit and a one gallon bath. All three units provide thermoregulator control, solid state circuitry, high-capacity pump, stainless steel immersion components and adjustable heater wattage. Details of these circulators are given in Bulletin Number 531.

*Fisher Scientific Co, 711 Forbes Avenue, Pittsburgh, PA 15219, USA.*

### ESR system

The Sterilin Gazette, Number 11, features news on the Accu-Tech ESR (Erythrocyte Sedimentation Rate) system, which is now available in the UK from Sterilin. There is an article on the 'Glassless Revolution' with details on the production of Petri dishes plus information on other products available from this company.

*Sterilin Ltd, 43-45 Broad Street, Teddington, Middlesex TW11 8QZ, UK.*

### Benzene and toluene analysis

A data sheet available from Techmation describes the analysis of benzene and toluene in petrol to ASTM D3606 using a Carle series S analytical gas chromatograph. Application number 159-A uses two columns connected by a backflush valve, and reduces interference from aliphatics. Separation of aromatics is based on their boiling points. The series S uses an inbuilt precision valve controller factory-set for each 159-A instrument. Valve operation is critical, and results in an initial peak representing non-aromatics below C9, followed by benzene, an internal standard, then toluene. Total analysis time is claimed to be six minutes from injection of a 2 μl sample.

*Techmation Ltd, 58 Edgware Way, Edgware, Middlesex, HA8 8JP, UK.*

### Interface system

A new publication from Bio-Rad Laboratories outlines the use of the CIRA 101 GC/IR interface, coupled with its solid sample accessory, and demonstrates the possibilities of the system by detailing the analysis of the solvent system used in a commercial stamp-pad ink. The experimental detail is fully described and specifies all the chromatographic conditions, together with a typical chromatogram and infra red spectra of the five components used in the solvent system. Concentrations of the components range from 1.2% water to 21% triethylene glycol.

*Bio-Rad Laboratories Ltd, Caxton Way, Holywell Industrial Estate, Watford, Herts, WD1 8RP, UK.*

### Equipment up-date

The current issue of Fisher Scientific's Laboratory Reporter (number 18:1) highlights the latest developments in laboratory hardware, software and technique. It features: the Jet-Clean washer —a programmable unit for cleaning most laboratory glassware; the coal analyser — a self-contained, microprocessor-controlled instrument that can analyse a batch of 80 samples for residual moisture, volatile matter, ash and fixed carbon; and a new microcentrifuge that can handle sixteen 250 μl to 1.5 ml tubes at speeds up to 13750 rpm. The issue also gives details on HPLC-grade water and newly formulated reagents for the sulphur analyser plus convenience supplies such as French curves, an adhesive cement and transparent storage boxes.

*Fisher Scientific Co, 711 Forbes Avenue, Pittsburgh, PA 15219, USA.*

### Waveform processing systems

Datalab has produced a leaflet about waveform processing systems. This features a photograph of the system and a definition of the concept of waveform processing. It explains that the essential elements are a transient recorder to capture analogue signals in digital form, and a backing store such as a magnetic tape recorder to store the digital records for subsequent recall and replay in analogue form. The leaflet also shows a 19 channel transient recorder, associated with a magnetic tape recorder and a CBM model 3032 computer. The features of a system without the computer are listed and then the additional facilities made available by the addition of the computer are set out. This is followed by three examples of typical displays by the computer.

*Data Laboratories Ltd, 28 Wates Way, Mitcham, Surrey, CR4 4HR, UK.*

### Clinical diagnostic products

Bio-Rad Laboratories have published a catalogue for their range of clinical diagnostic products. The catalogue details the company's complete range of test systems including radioassay systems for thyroid, anaemia and drug monitoring; chromatography techniques for catecholamines, porphyria, lead poisoning and diabetes management; enzyme systems for lipid chemistry; together with a complete range of human based sera and urine controls.

*Bio-Rad Laboratories Ltd, Caxton Way, Holywell Industrial Estate, Watford, Herts, WD1 8RP, UK.*

